# M2-like Macrophages-derived CCL17 Promotes Esophageal Squamous Cell Carcinoma Metastasis and Stemness *via* Activating CCR4-mediated ERK/PD-L1 Pathway

**DOI:** 10.2174/0115665240312877241010123403

**Published:** 2024-10-25

**Authors:** Chun Jin, Liangliang Lu, Jian Gao, Ling Chen

**Affiliations:** 1 Department of Thoracic Surgery, Changhai Hospital, Second Military Medical University (Naval Medical University), No.168 Changhai Road, Yangpu District, 200433, Shanghai, P. R. China;; 2 Department of Anesthesia, Changhai Hospital, Second Military Medical University (Naval Medical University), No.168 Changhai Road, Yangpu District, 200433, Shanghai, P. R. China;; 3 Department of Thoracic Surgery, Zhongshan Hospital, Fudan University, No. 180 Fenglin Road, Xuhui District, 200030, Shanghai, P. R. China

**Keywords:** TAMs, CCL17, CCR4, ERK/PD-L1 pathway, ESCC

## Abstract

**Background and objective:**

High morbidity, high mortality and poor prognosis of esophageal squamous cell carcinoma (ESCC) highlights the urgent need for novel therapeutic strategies against ESCC. The current study addresses the precise role of M2-like macrophages-derived CCL17 in ESCC progression and to thoroughly elucidate the intrinsic molecular mechanisms.

**Methods:**

In this work, for functional experiments, Eca109 cells cultivated in M2-CM were treated with anti-IgG (50 µg/ml) or anti-CCL17 (50 µg/ml) to expound the tumor-promoting effects of M2-like macrophage-derived CCL17 in ESCC. Moreover, for rescue experiments, Eca109 cells were treated with CCL17 (50 ng/ml) and/or CCR4 antagonist AZD2098 (20 µM) to probe whether CCL17 could influence the malignant behaviors including migration, invasion and stemness of ESCC cells *via* activating CCR4/ERK/PD-L1 pathway.

**Results:**

Markedly enhanced CCL17 secretion was observed in M2-like macrophages. CCL17 bound to CCR4 to activate ERK/PD-L1 signaling. M2-like macrophages-derived CCL17 facilitated ESCC cell migration and invasion and enhanced stemness characteristics of ESCC cells, which were partially reserved by AZD2098 treatment. The tumor-promoting effects of M2-like macrophages-derived CCL17 on ECSS was depended on the activation of CCR4/ERK/PD-L1 pathway.

**Conclusion:**

To conclude, M2-like macrophages-derived CCL17 could facilitate ESCC cell migration and invasion and enhance stemness characteristics of ESCC cells *via* activating CCR4/ERK/PD-L1 signaling.

## 1 INTRODUCTION

Esophageal cancer (EC), a common type of human digestive tract cancer, ranks seventh cancer morbidity and sixth cancer mortality with a total of 604,100 new cases and 544,076 EC-related deaths occurring in 2020 [1, 2]. Esophageal squamous cell carcinoma (ESCC) constituting approximately 85% of cases worldwide is the predominant histological type of EC [3]. According to the survey, the majority of patients with ESCC are diagnosed at the advanced stage owing to the lack of obvious clinical symptoms in early ESCC patients [4]. Nowadays, single application or combinations of surgery, chemotherapy, radiotherapy and molecular targeted therapy are available as therapeutic strategies for ESCC [5]. However, the early metastasis and high probability of recurrence greatly contribute to the poor prognosis of ESCC patients [6].

Tumor microenvironment (TME) compositions including various immune cells and nonimmune stromal cells play determinant roles in tumor growth or decline [7]. Tumor-associated macrophages (TAMs), the most abundant immune cells of TME, have attracted enormous attention as they are adverse prognostic factors for various types of cancers [8, 9]. TAMs facilitate tumor malignancy predominantly through release of diverse factors, such as cytokines, chemokines and growth factors [10]. As reported in the literature, CCL17, CCL18 and CCL22, characteristic C-C chemokines released by TAMs, are closely associated with the malignant progression and poor prognosis of a series of cancers [11]. TAMs secreted CCL17 can promote tumor growth and invasion of human pituitary adenoma and enhance susceptibility to postoperative recurrence [12]. TAMs secreted CCL17 can confer colorectal cancer 5-fluorouracil resistance by activating PI3K/AKT pathway [13]. TAMs secreted CCL18 can promote cell proliferation to facilitate ESCC progression *via* activating JAK2/STAT3 pathway, leading to poor prognosis of ESCC [14]. TAMs secreted CCL22 can promote ESCC cell proliferation, migration and invasion to facilitate tumor progression *via* activating FAK signaling [15]. Nevertheless, the tumor-promoting effects of TAMs-derived CCL17 on ESCC development remains uncertain till now.

It is noteworthy that the elevated expression level of CCL17 is accompanied by the higher expression of its receptor C-C motif chemokine receptor 4 (CCR4), specifically within macrophage populations [16]. Abundant evidences indicate that CCL17 can perform biological functions through interacting with CCR4 in a series of diseases, including tumor-promoting effects on hepatocellular carcinoma and bladder cancer [17-22]. Significantly, CCR4 activated by CCL22 can initiate assembly of CCR4/DGKα/FAK protein complex to promote CCL22-induced ESCC metastasis [12]. In addition, CCR4 has been reported to aggravate tumor malignancy and facilitate tumor metastasis through activation of ERK signaling in bladder cancer, hepatocellular carcinoma and colorectal cancer [20, 23, 24]. As evidenced by numerous researches, ERK can function as an oncogene to promote ESCC malignant progression and inhibition of ERK signaling can strengthen the efficacy of radiochemotherapy in ESCC therapies [25-28]. Additionally, it is well-documented that ERK can exert the tumor-promoting role in a lot of malignancies through upregulation of PD-L1 expression, such as ESCC, breast cancer, colon cancer, intrahepatic cholangiocarcinoma, non-small cell lung carcinoma and gastric cancer [29-34].

M2-like macrophages are the primary expression form of TAMs [35]. In the present study, it was hypothesized that M2-like macrophages-derived CCL17 could facilitate ESCC malignant progression through activating CCR4 signaling to mediate ERK/PD‑L1 pathway. The tumor-promoting role of M2-like macrophages-derived CCL17 in ESCC was investigated from these aspects: cancer cell migratory ability, cancer cell invasive ability and cancer cell stemness characteristics. Moreover, whether M2-like macrophages-derived CCL17 could exert its tumor-promoting effects *via* CCR4-mediated ERK/PD‑L1 signaling was thoroughly elucidated. Collectively, results of our work indicated that M2-like macrophages-derived CCL17 might be a key driver of ESCC metastasis and recurrence.

## 2 MATERIALS AND METHODS

### 2.1 Cell Culture

Human ESCC cell line Eca109 (Cellverse, Shanghai, China) were cultured in DMEM (Hyclone, UT, USA) supplemented with 10% FBS (Invitrogen, CA, USA) and 1% antibiotic mixture at 37 °C in a humidified atmosphere with 5% CO_2_. Human monocytic cell line THP-1 (Cellverse, Shanghai, China) were cultured in RPMI-1640 complete medium (Hyclone, UT, USA) at 37 °C in a humidified atmosphere with 5% CO_2_.

### 2.2 Induction of M2-like Macrophages [36]

M2 polarization of THP-1 cells was induced by PMA and IL-4. Briefly, THP-1 cells were first treated with 100 nM PMA (Sigma-Aldrich, MO, USA) for 48 h, followed by treatment with 20 ng/ml IL-4 (Invitrogen, CA, USA) for another 24 h . After that, CCL17 level in cell culture supernatant was scheduled for ELISA detection.

### 2.3 Collection of Conditioned Medium (CM) [12]

M2-like macrophages (5×10^5^ cells/well) were cultured in 6-well plates in serum-starved RPMI-1640 medium for 24 h. The cell culture supernatants gathered by centrifugation was passed through a 0.22-μm sterile filter and then collected as M2-CM.

### 2.4 Cell Treatment

To elucidate the precise role of M2-like macrophage-derived CCL17 in ESCC progression, Eca109 cells cultivated in M2-CM were treated with anti-IgG (50 µg/ml) or anti-CCL17 (50 µg/ml; R&D Systems, MN, USA) [37].

To probe whether CCL17 could influence the malignant behaviors of ESCC *via* CCR4/ERK/PD-L1, Eca109 cells were treated with CCL17 (50 ng/ml; R&D Systems, MN, USA) and/or CCR4 antagonist AZD2098 (20 µM; MedChem Express, NJ, USA) [12].

### 2.5 Wound Healing Assay

Eca109 cells grown to 90% confluency on culture plates were scratched vertically with a sterile pipette tip (200 µl) to create the “wound”, followed by the removal of scratched cells by PBS rinse. Next, Eca109 cells were supplemented with serum-starved DMEM medium for 24 h incubation at 37 ˚C. Images of the wounded area were observed and photographed at 0 and 24 h after wounding under a light microscope.

### 2.6 Transwell Assay

Eca109 cells re-suspended in serum-starved DMEM medium were seeded into the upper layer of transwell chamber pre-coated with matrigel and DMEM complete medium was supplemented into the lower layer of transwell chamber as a chemoattractant. Post 24 h of incubation, non-invasive cells were gently removed using cotton swabs and the invasive cells in the lower chamber were fixed in 4% paraformaldehyde, colored with 0.1% crystal violet and then photographed and quantitatively counted under a light microscope.

### 2.7 Spheroids Formation Assay

Eca109 cells (5×10^3^ cells/well) seeded in 6-well ultra-low attachment plates were cultured in serum-starved DMEM medium supplemented with 20 ng/ml epidermal growth factor (EGF), 20 μl/ml B27 supplement and 20 ng/ml basic fibroblast growth factor (bFGF). After 7 days of incubation, the number of tumor spheroids (diameter > 100 μm) were counted under a light microscope.

### 2.8 Immunofluorescence Staining

Eca109 cells were subjected to 4% paraformaldehyde at room temperature for 30 min for fixation and 0.1% Triton X-100 at 4 °C for 20 min for permeabilization. After blocking in 5% BSA at room temperature for 1 h, Eca109 cells were probed with primary antibodies as follows: anti-CD206 (Abcam, ab64693, 1:1000), anti-Arg1 (Abcam, ab96183, 1:1000), anti-SOX2 (Abcam, ab97959, 1:1000) and anti-CCR4 (Invitrogen, PA1-41155, 1:2000) at 4 ˚C overnight, followed by incubation with secondary antibody conjugated with Alexa Fluor^®^ 488 (Abcam, ab150077, 1:1000) or Alexa Fluor^®^ 594 (Abcam, ab150080, 1:1000) at room temperature for 1 h away from the light. Next, Eca109 cells were counterstained with DAPI and then photographed under a fluorescence microscope.

### 2.9 Western Blot Assay

The concentration of total protein extracted from Eca109 cells was measured using BCA method. After separation by SDS-PAGE according to molecular weight, proteins were transferred onto PVDF membranes, followed by sealing in 5% BSA to block nonspecific binding proteins. After that, membranes were probed with primary antibodies as follows: anti-MMP2 (Abcam, ab92536, 1:1000), anti-MMP9 (Abcam, ab76003, 1:1000), anti-SOX2 (Abcam, ab92494, 1:1000), anti-OCT4 (Abcam, ab19857, 1:1000), anti-Nanog (Abcam, ab109250, 1:1000), anti-CCR4 (Abcam, ab315446, 1:1000), anti-p-ERK (Abcam, ab201015, 1:1000), anti-ERK (Abcam, ab184699, 1:10000) and anti-PD-L1 (Abcam, ab213524, 1:1000) at 4 °C overnight, followed by incubation with HRP-conjugated secondary antibody (Abcam, ab205718, 1:50000) at room temperature for 1 h. β-actin served as the internal reference. Band densitometry analysis was performed using Image J software.

### 2.10 Statistical Analysis

Statistical differences among multiple groups or two groups were analyzed by one-way ANOVA followed by Tukey’s post hoc test or unpaired Student’s t-test, respectively. Data of three independent experiments were expressed as mean ±SD. Differences were considered statistically significant when P < 0.05.

## 3 RESULTS

### 3.1 Enhanced CCL17 Secretion in M2-like Macrophages

**Fig. (1):**
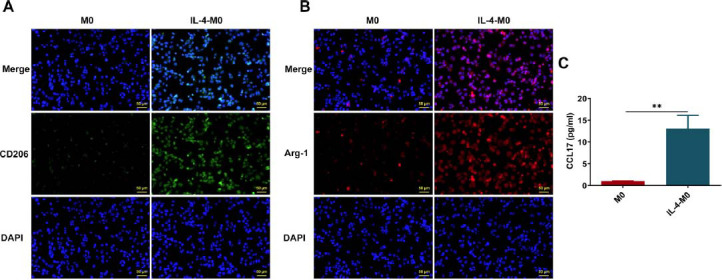
**Enhanced secretion of CCL17 in M2-like macrophages.** M2 polarization of THP-1 cells was induced by PMA and IL-4. THP-1 cells were first treated with 100 nM PMA for 48 h, followed by treatment with 20 ng/ml IL-4 for another 24 h. (**A**) Immunofluorescence staining for detection of CD206 expression (magnification, ×200). (**B**) Immunofluorescence staining for detection of Arg-1 expression (magnification, ×200). (**C**) ELISA for detection of CCL17 secretion. ** *p*<0.01.

M2-like macrophages are activated by Th2 cytokine IL-4. As presented by immunofluorescence staining, extremely elevated expressions of M2-like macrophage-associated markers (CD206 and Arg-1) indicated that IL-4 treatment induced M2-like macrophage polarization (Fig. **1A**, **B**). Interestingly, markedly enhanced CCL17 secretion was observed in M2-like macrophages (Fig. **1C**).

### 3.2 M2-like Macrophages-derived CCL17 Facilitates ESCC Cell Migration and Invasion

**Fig. (2):**
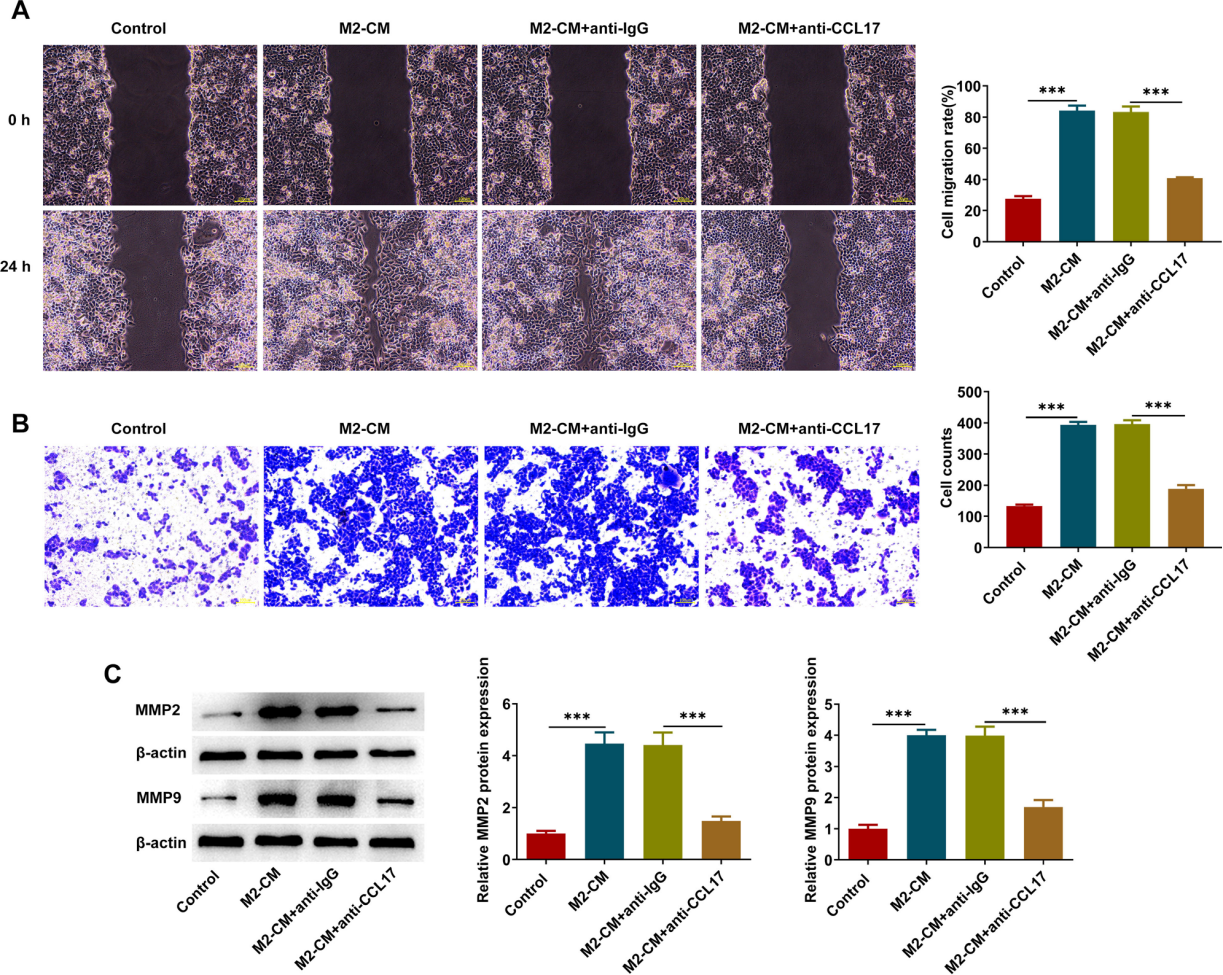
**M2-like macrophages-derived CCL17 facilitates ESCC cell migration and invasion.** Eca109 cells cultivated in M2-CM were treated with anti-IgG or anti-CCL17. (**A**) Wound healing assay for determination of cell migratory ability (magnification, ×100). (**B**) Transwell assay for determination of cell invasive ability (magnification, ×100). (**C**) Western blot analysis for detection of MMP2 and MMP9 protein expressions. *** *p*<0.001.

In this work, Eca109 cells cultivated in M2-CM were treated with anti-IgG or anti-CCL17 in order to investigate the precise influences of M2-like macrophages-derived CCL17 on the malignant behaviors of ESCC cells. Results of wound healing and transwell assays indicated that M2-like macrophages-derived CCL17 enhanced the migratory and invasive capabilities of Eca109 cells, which were partially reserved by anti-CCL17 treatment (Fig. **2A**, **B**). Besides, reduced expressions of metastasis-associated proteins MMP2 and MMP9 upon anti-CCL17 treatment also indicated that the promoting effects of M2-like macrophages-derived CCL17 on ESCC cell migration and invasion were partially abolished by anti-CCL17 treatment (Fig. **2C**).

### 3.3 M2-like Macrophages-derived CCL17 Enhances Stemness Characteristics of ESCC Cells

**Fig. (3):**
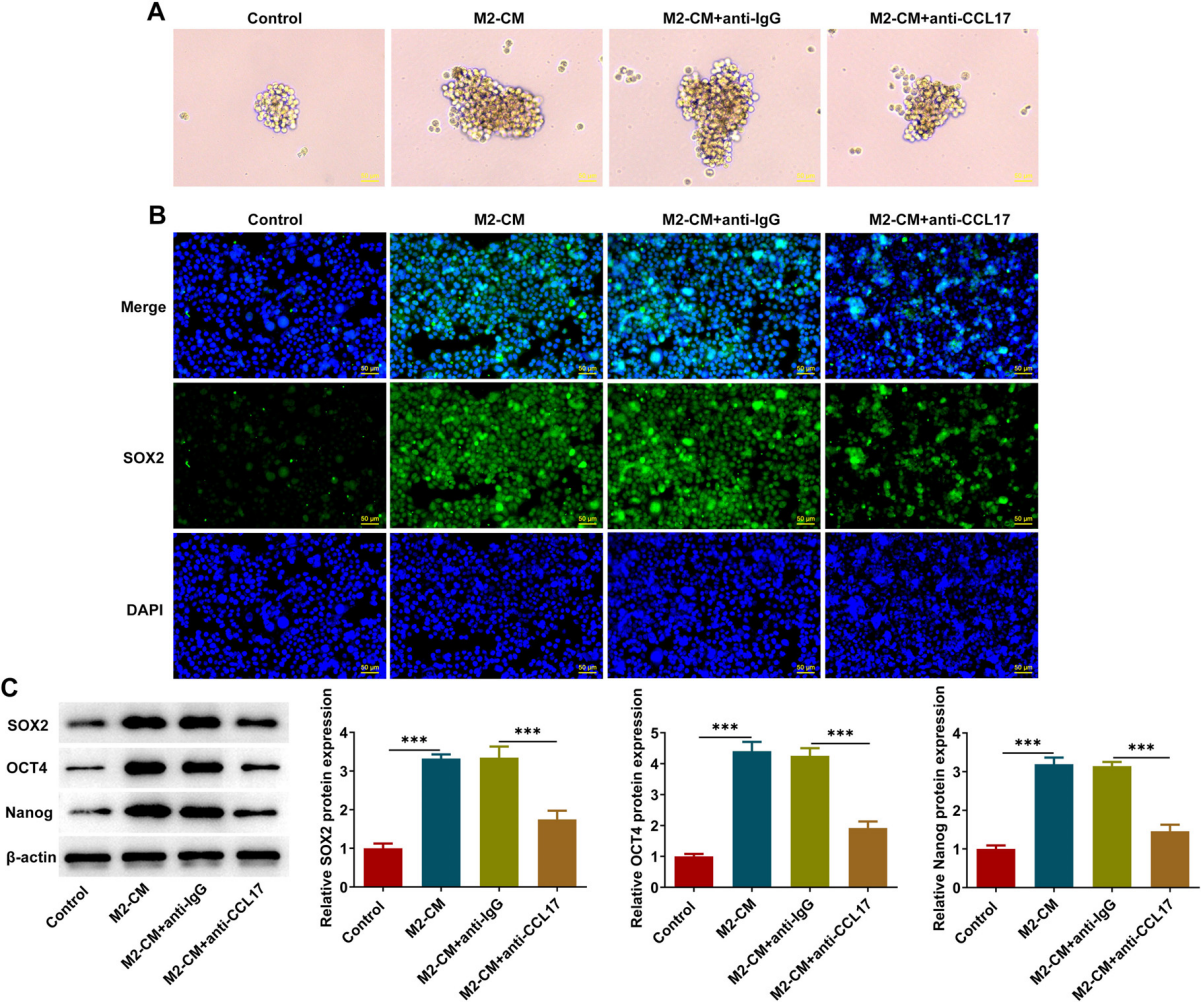
**M2-like macrophages-derived CCL17 enhances stemness characteristics of ESCC cells.** Eca109 cells cultivated in M2-CM were treated with anti-IgG or anti-CCL17. (**A**) Sphere formation assay for determination of sphere formation efficiency (magnification, ×200). (**B**) Immunofluorescence staining for detection of SOX2 expression (magnification, ×200). (**C**) Western blot analysis for detection of SOX2, OCT4 and Nanog protein expressions. *** *p*<0.001.

Sphere formation assay is a prominent method to evaluate the stemness of tumor cells. It was observed that M2-like macrophages-derived CCL17 promoted sphere formation of Eca109 cells, which were partially reserved by anti-CCL17 treatment (Fig. **3A**). Additionally, expressions of stemness-associated proteins SOX2, OCT4 and Nanog were detected. M2-like macrophages-derived CCL17 strengthened SOX2 fluorescence as well as elevated the protein expressions of SOX2, OCT4 and Nanog, which were partially reserved by anti-CCL17 treatment (Fig. **3B**, **C**). These results indicated that the enhancing effects of M2-like macrophages-derived CCL17 on stemness characteristics of ESCC cells were partially abolished by anti-CCL17 treatment.

### 3.4 CCL17 Binds to CCR4 to Activate ERK/PD-L1 Signaling

**Fig. (4):**
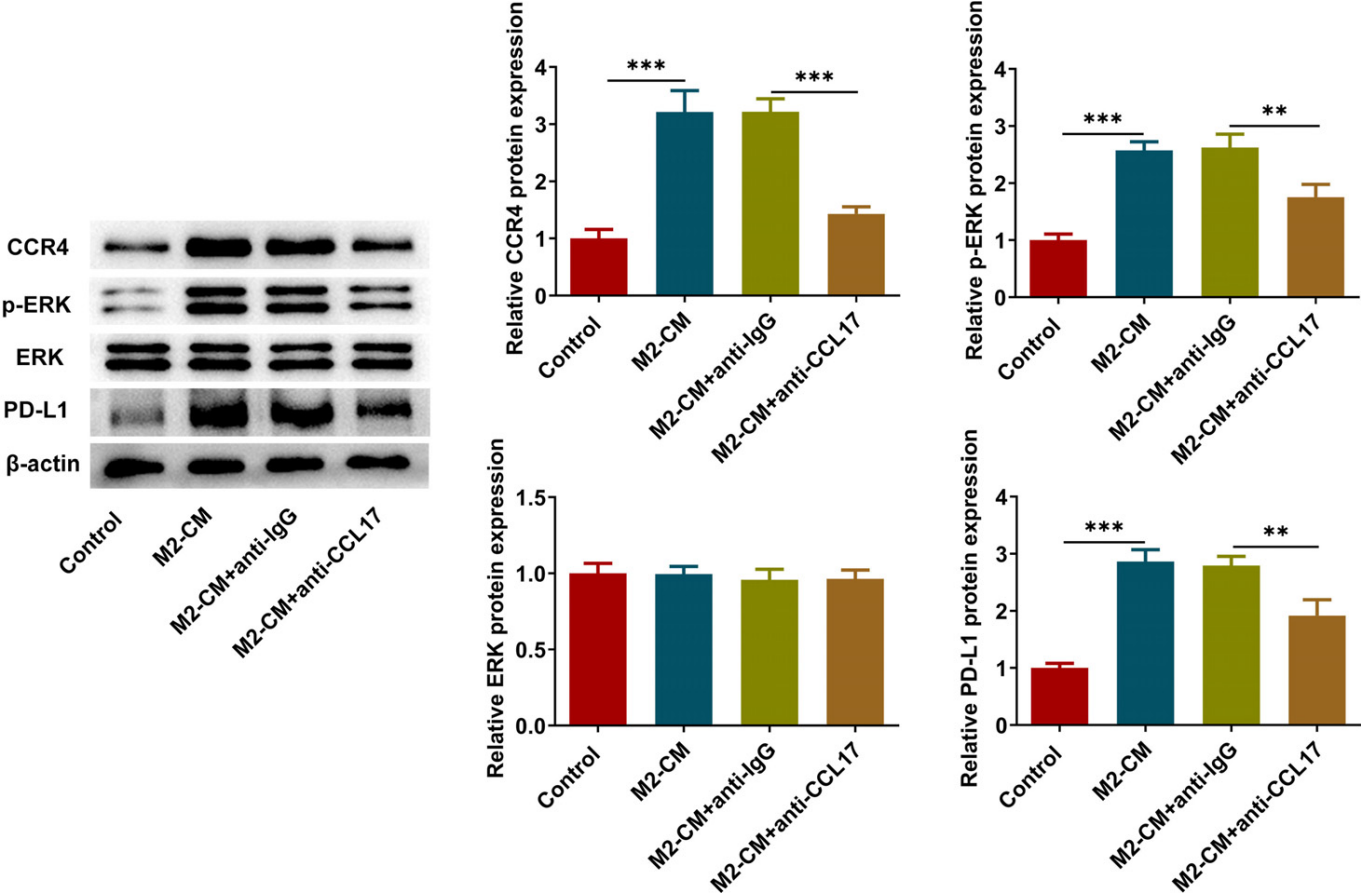
**CCL17 binds to CCR4 to activate ERK/PD-L1 signaling.** Eca109 cells cultivated in M2-CM were treated with anti-IgG or anti-CCL17. Western blot analysis for detection of CCR4, p-ERK, ERK and PD-L1 protein expressions. ** *p*<0.01, *** *p*<0.001.

**Fig. (5):**
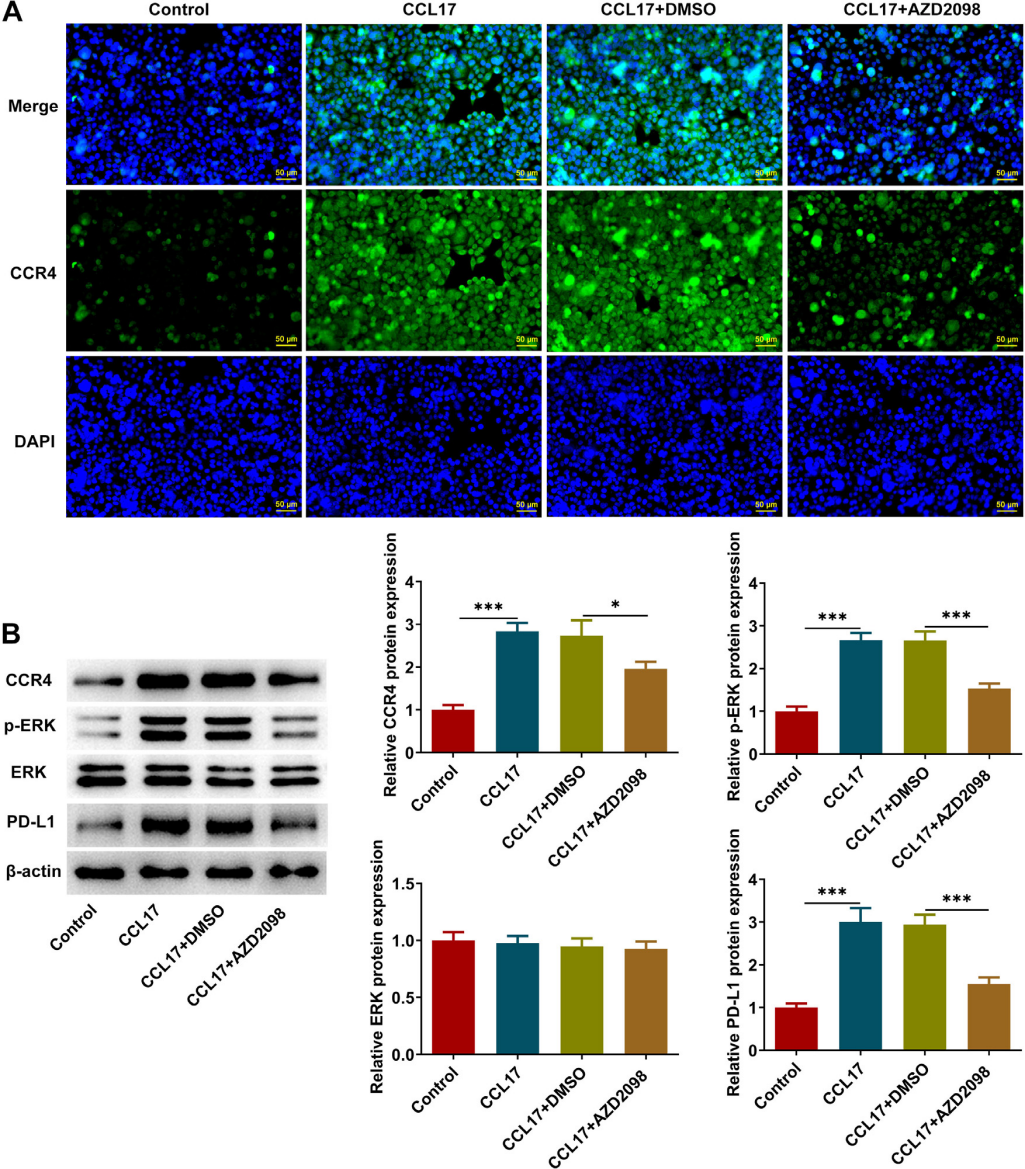
**CCL17 binds to CCR4 to activate ERK/PD-L1 signaling.** Eca109 cells were treated with CCL17 protein or co-treated with CCL17 protein and CCR4 antagonist AZD2098. (**A**) Immunofluorescence staining for detection of CCR4 expression (magnification, ×200). (**B**) Western blot analysis for detection of CCR4, p-ERK, ERK and PD-L1 protein expressions. * *p*<0.05, *** *p*<0.001.

Based on findings above, this work sought to identify the potential downstream signaling of CCL17 in order to deeply explore the intrinsic molecular mechanisms. M2-like macrophages-derived CCL17 elevated the protein expressions of CCR4, p-ERK and PD-L1, which were partially reversed by anti-CCL17 treatment (Fig. **4**). This phenomenon indicated that M2-like macrophages-derived CCL17 activated CCR4/ERK/PD-L1 signaling and anti-CCL17 treatment inactivated CCR4/ERK/PD-L1 signaling. Furthermore, Eca109 cells were treated with exogenous CCL17 or co-treated with exogenous CCL17 and CCR4 antagonist AZD2098 in order to further expound the oncogenic role of CCL17 in ESCC and to thoroughly elucidate the underlying mechanisms. CCL17 treatment strengthened CCR4 fluorescence and elevated the protein expressions of CCR4, p-ERK and PD-L1, and these regulating effects were partially abolished by AZD2098 treatment (Fig. **5A**, **B**). In a word, CCL17 may bind to CCR4 to activate ERK/PD-L1 pathway.

### 3.5 CCL17 Facilitates ESCC Cell Migration and Invasion *via* Activating CCR4/ERK/PD-L1 Signaling

**Fig. (6):**
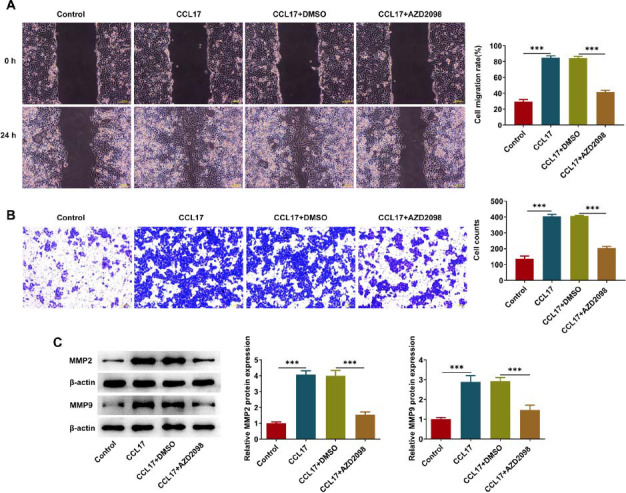
**CCL17 facilitates ESCC cell migration and invasion *via* activating CCR4/ERK/PD-L1 signaling.** Eca109 cells were treated with CCL17 protein or co-treated with CCL17 protein and CCR4 antagonist AZD2098. (**A**) Wound healing assay for determination of cell migratory ability (magnification, ×100). (**B**) Transwell assay for determination of cell invasive ability (magnification, ×100). (**C**) Western blot analysis for detection of MMP2 and MMP9 protein expressions. *** *p*<0.001.

CCL17 treatment enhanced the migratory and invasive capabilities of Eca109 cells, which were partially reserved by AZD2098 treatment (Fig. **6A**, **B**). Besides, reduced expressions of MMP2 and MMP9 upon AZD2098 treatment also indicated that the promoting effects of CCL17 on ESCC cell migration and invasion were partially abolished by AZD2098 treatment (Fig. **6C**). To sum up, CCL17 may facilitate ESCC cell migration and invasion *via* binding to CCR4 to activate ERK/PD-L1 pathway.

### 3.6 CCL17 Enhances Stemness Characteristics of ESCC Cells *via* Activating CCR4/ERK/PD-L1 Signaling

**Fig. (7):**
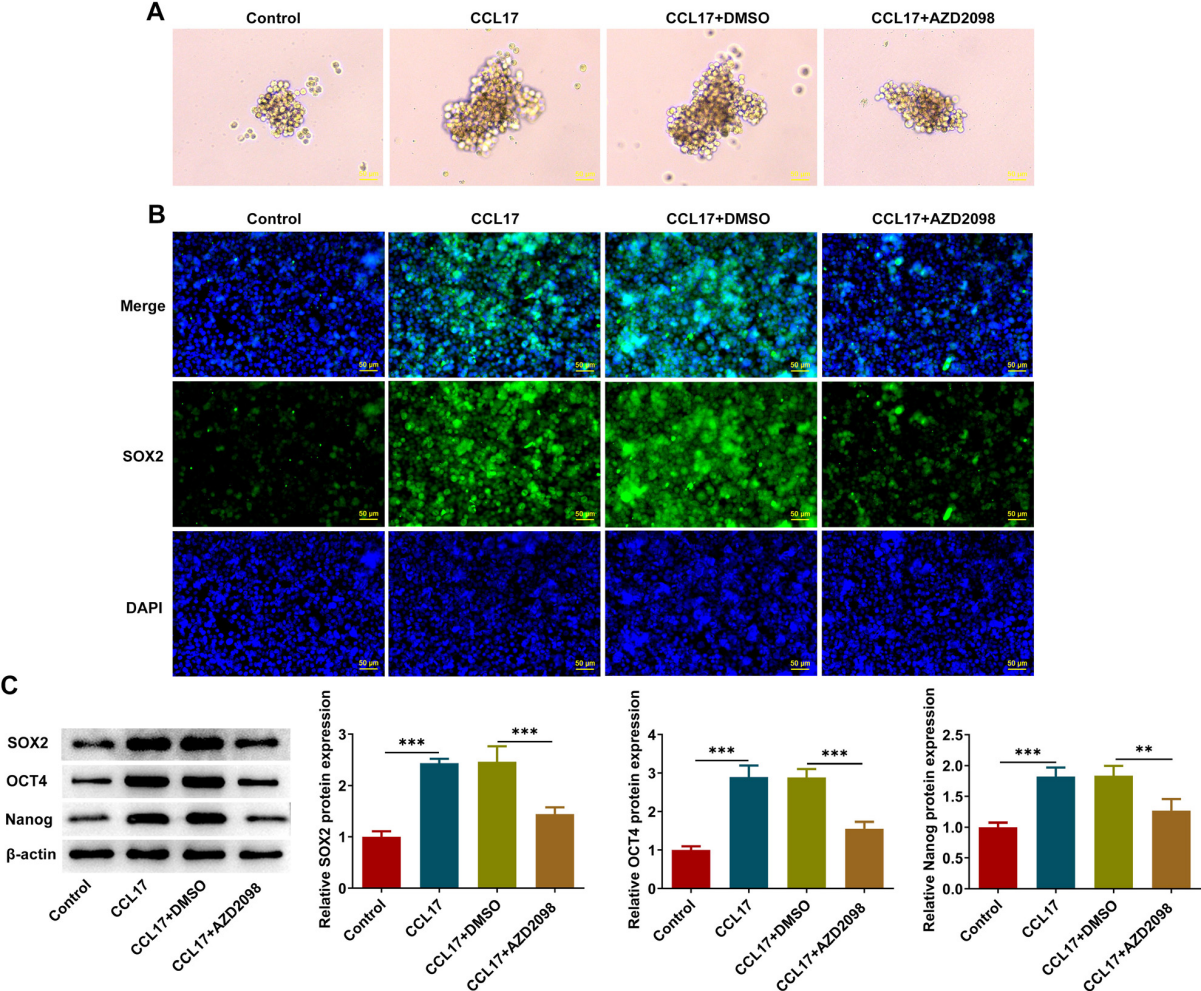
**CCL17 enhances stemness characteristics of ESCC cells *via* activating CCR4/ERK/PD-L1 signaling.** Eca109 cells were treated with CCL17 protein or co-treated with CCL17 protein and CCR4 antagonist AZD2098. (**A**) Sphere formation assay for determination of sphere formation efficiency (magnification, ×200). (**B**) Immunofluorescence staining for detection of SOX2 expression (magnification, ×200). (**C**) Western blot analysis for detection of SOX2, OCT4 and Nanog protein expressions. ** *p*<0.01, *** *p*<0.001.

CCL17 treatment promoted sphere formation of Eca109 cells, which were partially reserved by AZD2098 treatment (Fig. **7A**). Additionally, CCL17 treatment strengthened SOX2 fluorescence as well as elevated the protein expressions of SOX2, OCT4 and Nanog, which were partially reserved by AZD2098 treatment (Fig. **7B**, **C**). To sum up, CCL17 may enhance stemness characteristics of ESCC cells *via* binding to CCR4 to activate ERK/PD-L1 pathway.

## 4 DISCUSSION

Despite advances in diagnosis and multimodal therapy, ESCC treatment remains challenging due to tumor metastasis and recurrence [38, 39]. This current work addresses the tumor-promoting effects of M2-like macrophages-derived CCL17 on the migration, invasion and stemness characteristics of ESCC cells and to thoroughly elucidate the intrinsic molecular mechanisms, aiming to develop promising targets and drugs for ESCC therapies.

Interaction among ESCC and different types of cells in TME determines ESCC development [40]. TAMs, infiltrated inflammatory cells in TME, can facilitate malignant tumor initiation and progression. Meanwhile, clinical studies show that TAMs density has an inseparable correlation with poor prognosis in patients with solid tumors, including ESCC [41, 42]. During immune responses, macrophages secrete a variety of cytokines, chemokines as well as other immune mediators to perform intracellular communication [43]. In our work, it was verified that M2-like macrophages-derived CCL17 facilitated ESCC cell migration and invasion and enhanced stemness characteristics of ESCC cells.

CCR4 is a high-affinity receptor for CCL17. CCL17 can activate CCR4 to create Th2 dominant condition to modify sensitizing reagents as well as irritant reagents caused inflammation in the lesional skin [21]. CCL17 can promote fibroblast activation and tissue fibrosis *via* interacting with CCR4 on fibroblasts to activate TGF-β/Smad pathway [17]. CCL17-CCR4 axis can contribute to vitiligo progression in mice by facilitating T cell migrating to skin [18]. CCL17-CCR4 interactions can impair the pulmonary antifungal response against A. fumigatus in neutropenic mice [19]. CCL17-CCR4 axis can promote hepatocellular carcinoma progression and increase resistance to sorafenib by enhancing the migratory activities of macrophage and Treg cells [22]. CCL17-CCR4 axis can promote bladder cancer metastasis *via* activating ERK/MMP13 pathway [20]. Besides, literature reports that CCR4 can aggravate hepatocellular carcinoma malignancy and facilitate hepatocellular carcinoma cell metastasis *via* activating ERK/AKT/MMP2 pathway [24] and CCR4 can promote colorectal cancer metastasis *via* activating ERK/NF-κB/MMP13 pathway [23]. What’s more, literature reports that PPP1r18 can promote ESCC tumor progression *via* activating calcineurin-mediated ERK pathway [26]. Cysteine protease inhibitor 1 can promote ESCC metastasis *via* activating the oxidative phosphorylation/MEK/ERK axis [27]. NSG1 overexpression can promote ESCC cell EMT through activation of ERK pathway [25]. The therapeutic efficacy of both cisplatin and radiotherapy in ESCC can be enhanced by blocking MAPK/ERK pathway [28]. TRAIL overexpression can upregulate PD-L1 expression depending on activation of ERK/STAT3 pathway, thereby promoting ESCC progression [31]. Besides, knockdown of ERK can increase TRAIL-induced breast cancer cell death through decreasing PD-L1 expression [29]. ERK inhibitor PD98059 can suppress alphavbeta6-induced upregulation of PD-L1 expression to inhibit immune escape of colon cancer [30]. ERK signaling inhibition can downregulate PD-L1 expression through the autophagy pathway to induce the apoptosis of intrahepatic cholangiocarcinoma [32]. ERK inhibition can downregulate PD-L1 expression to enhance the efficacy of PD-1 blockage against non-small cell lung carcinoma *in vitro* and *in vivo* models [33]. Gastric cancer-derived exosomes can promote pulmonary metastasis by activating ERK signaling to facilitate PD-L1 expression [34]. In our work, it was verified that CCL17 bound to CCR4 activates ERK/PD-L1 signaling. CCL17 facilitated ESCC cell migration and invasion and enhanced stemness characteristics of ESCC cells, which were partially reserved by AZD2098 treatment. The tumor-promoting effects of M2-like macrophages-derived CCL17 on the malignant behaviors of ECSS was depended on the activation of CCR4/ERK/PD-L1 pathway.

## CONCLUSION

The present study illustrated for the first time the crosstalk between ESCC and M2-like macrophages-derived CCL17. M2-like macrophages-derived CCL17 could facilitate ESCC cell migration and invasion and enhance stemness characteristics of ESCC cells *via* activating CCR4/ERK/PD-L1 signaling. The current work provided convincing evidence for the oncogenic role of M2-like macrophages-derived CCL17 in ESCC progression, which may help to facilitate the development of therapeutic strategies for ESCC.

## Data Availability

The analyzed datasets during the present study are available from the corresponding author on reasonable request.

## LIST OF ABBREVIATIONS

DAPI: 4',6-diamidino-2-phenylindole
ANOVA: Analysis of Variance
Arg-1: Arginase-1
bFGF: Basic fibroblast growth factor
BCA: Bicinchoninic acid
BSA: Bovine serum albumin
CCL17: C-C motif chemokine ligand 17
CCR4: C-C motif chemokine receptor 4
CM: Conditioned Medium
DMEM: Dulbecco's Modified Eagle Medium
ELISA: Enzyme linked immunosorbent assay
EGF: Epidermal growth factor
EMT: Epithelial-mesenchymal transition
EC: Esophageal cancer
ESCC: Esophageal squamous cell carcinoma
ERK: Extracellular signal-regulated kinase
FBS: Fetal bovine serum
HRP: Horseradish peroxidase
IL-4: Interleukin 4
MMP2: Matrix metalloproteinase 2
MMP9: Matrix metalloproteinase 9
OCT-4: Octamer-binding transcription factor 4
PMA: Phorbol 12-myristate 13-acetate
PVDF: Polyvinylidene fluoride
PD-L1: Programmed death ligand 1
RPMI: Roswell Park Memorial Institute
SDS-PAGE: Sodium dodecyl sulfate polyacrylamide gel electrophoresis
SOX2: SRY-box transcription factor 2
SD: Standard deviation
TME: Tumor microenvironment
TAMs: Tumor-associated macrophages
